# Adenosarcoma of Uterus-Rare Biphasic Malignant Tumor: A Case Report

**DOI:** 10.31729/jnma.5373

**Published:** 2021-02-28

**Authors:** Dilasma Ghartimagar, Binaya Shrestha, Arnab Ghosh, Manish Kiran Shrestha, Junu Shrestha, Sushma Thapa, Om Prakash Talwar

**Affiliations:** 1 Department of Pathology, Manipal College of Medical Sciences, Pokhara, Nepal; 2 Department of Radiology, Charak Memorial Hospital, Pokhara, Nepal; 3 Department of Gynecology and Obstetrics, Manipal College of Medical Sciences, Pokhara, Nepal

**Keywords:** *adenosarcoma*, *biphasic*, *mesenchymal*, *uterus*

## Abstract

Uterine adenosarcoma is a rare variant of mixed Mullerian tumors comprised of neoplastic glands with the benign appearance and sarcomatous stroma. The epithelium most often consists of endometrium-like cells, while the sarcomatous component usually shows low-grade homologous uterine sarcoma. These tumors present as a pelvic mass or an enlarged uterus with abnormal vaginal bleeding. Here, we present a case of 61 years old postmenopausal female patient with chief complaints of excessive vaginal bleeding and urine retention.

## INTRODUCTION

Adenosarcoma of the endometrium is a rare biphasic malignant mesenchymal tumor composed of a benign endometrial glandular component and a malignant but generally low grade endometrial stromal component. Predisposing factors attributed to adenosarcoma are a history of pelvic radiation, breast cancer, hypertension, obesity, and treatment history with tamoxifen.^1^

## CASE REPORT

A 61-year-old female patient came with chief complaints of intermittent, excessive vaginal bleeding for two months. She also complained of vaginal discharge along with burning micturition and retention of urine for two days.

She had menopause five years back and uterine prolapse for one year. There was no history of hypertension or diabetes mellitus. Her general and systemic examinations were within the normal limit, but the per speculum examination revealed a polypoidal mass into the vagina, and the mass was protruding from the cervix.

Ultrasonography of abdomen and pelvis revealed bulky uterus measuring 13.7 x 9.3 x 5.5 cm with thickened endometrium and large heterogeneous mass in the cervix. MRI pelvis revealed large heterogeneously enhancing lesion with cystic areas involving cervix measuring 14 x 8 x 8.5 cm. The lesion was extending up to the left lateral uterine wall displacing the thickened endometrium (14.8 mm) to the right. An inferior lesion was reaching up to the vaginal introitus. No parametrial infiltration was seen. MRI impression was given as carcinoma cervix.

During surgery, a huge submucosal fibroid extending up to the vagina was noticed. Adhesions were present over the posterior surface of the uterus and to the bowel mucosa. Mass including the uterus and bilateral adnexa could not be removed intact. Tissues were sent for histopathological examination with a provisional diagnosis of carcinoma cervix.

**Figure 1a.**
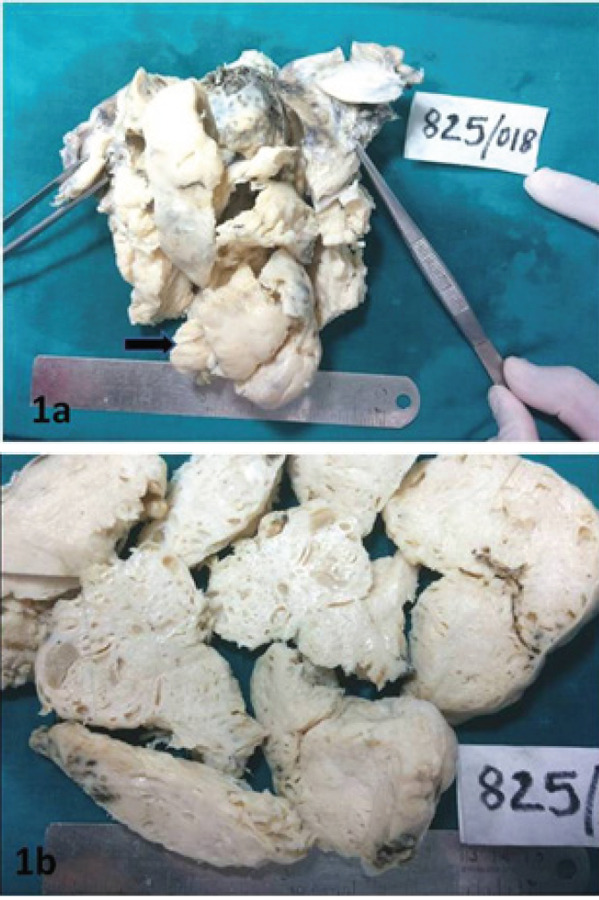
Tumor tissues sent in multiple bits with a large grey white mass (arrow) protruding from the cervical canal. Figure 1b. Cut surface of the tumor tissues showed multiple cystically dilated spaces within the tumor mass.

On gross examination, the uterine mass was received in multiple bits and altogether weighed 400 grams and measured 9.5 x 7.5 x 3 cm. The endometrial cavity showed multiple exophytic masses extending into the endometrial cavity, the largest measuring 2 x 1 cm. Friable irregular areas measuring 4 x 4.5 cm was noted protruding from the cervix (Figure 1a). The cut surface of the mass showed multiple cystically dilated spaces within the tumor mass (Figure 1b). Bilateral adnexa appeared unremarkable grossly. Cervix was sent separately, which showed single dark brown to pale white friable tissue measuring 0.7 x 0.5 cm.

**Figure 2a.**
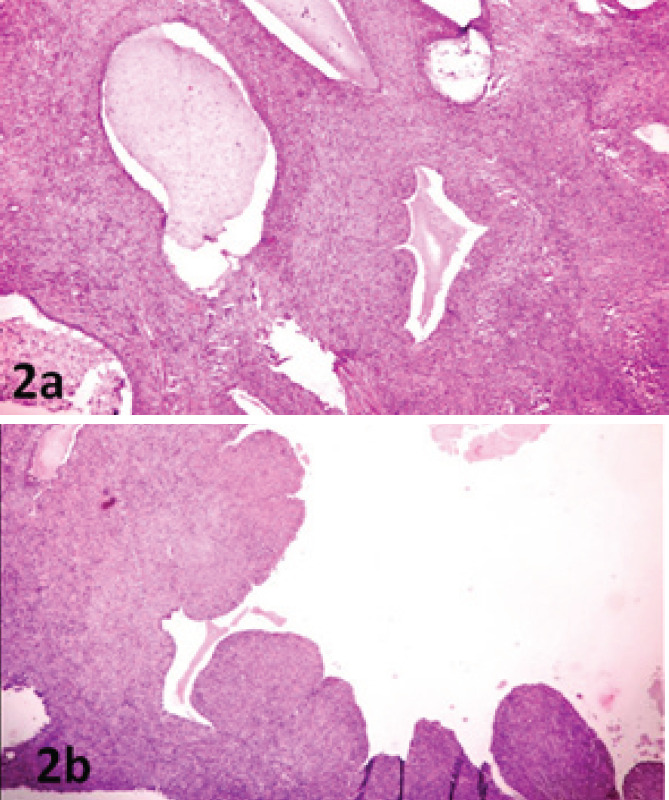
Microscopic picture showed biphasic tumor with proliferation of glands and stroma. Glands were cystically dilated and show luminal secretion (H&E x 400). Figure 2b. Microscopic picture of tumor showing polypoidal fronds of stroma projecting into cystic gland giving phyllodes-like appearance (H&E x 400).

**Figure 3a.**
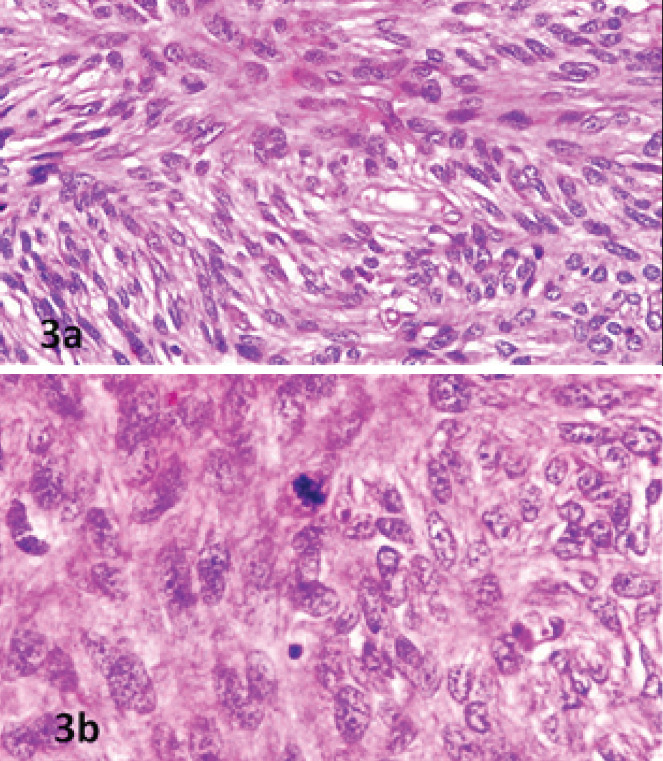
High power view showing malignant stromal cells arranged in fascicles. (H&E x 1000) Figure 3b. Malignant stromal cells with atypical mitosis (H&E x 1000).

Microscopic examination showed biphasic proliferation of tumor with glands and stroma. The stroma was cellular with mild to moderate atypia and was seen cuffing around the glands. The glands were distributed throughout the tumor and were lined by columnar epithelium. Glands were cystically dilated with luminal secretion (Figure 2a). The focal area showed papillary to polypoid fronds of stroma projecting into cystic glands giving a phyllodes-like appearance (Figure 2b). Some areas showed slit-like spaces with cuboidal lining. The stroma was arranged in bundles, fascicles, and storiform patterns with hypo and hypercellular areas. Hypercellular areas were mainly present around the glands. The cells were spindle-shaped with mild to moderate nuclear pleomorphism with prominent nucleoli (Figure 3a). Mitotic figures were 2-3/10 hpf (Figure 3b). Focal areas of necrosis were also noted. Cervical biopsy showed dense inflammation with areas of hemorrhage, but the cervical lining was not identified. Histopathological diagnosis was given as adenosarcoma.

## DISCUSSION

Adenosarcoma is a rare variant of mixed Mullerian tumors, consisting of neoplastic glands with a benign appearance and a sarcomatous stroma.^2,3^ It occurs in women of all ages.^1^ The majority occur in postmenopausal women, but about 30% are found in premenopausal patients, including adolescents. The median age for the tumor is 50-59 years.^4^ Here, we report a case of adenosarcoma in 61 years old postmenopausal female who presented with abnormal vaginal bleeding, discharge, urinary retention, and cervical growth.

Extrauterine adenosarcoma occurs in younger women and is more aggressive than its uterine counterpart. It most often occurs in the endometrium but is also found in the cervix and in extrauterine pelvic locations, such as the fallopian tube, ovary, and para ovarian tissues.^1^

Association of adenosarcoma with obesity or hypertension is not seen.^1^ Risk factors for uterine adenosarcoma include unopposed estrogen stimulation,^5^ long-term oral contraceptive use^6^ and prolonged use of tamoxifen for breast cancer.^7,8^ Our patient had none of these risk factors. These tumors can present as a pelvic mass (37%), uterine polyp (22%), or an enlarged uterus (22%).^9^ Abnormal vaginal bleeding is the most common presenting symptom. Vaginal discharge, pain, nonspecific urinary symptoms, a palpable pelvic mass, and a tumor protruding from the cervix are other common signs and symptoms.^1^ There may be a history of the uterine polyp.^4^

At low power, the architecture is of a phyllodes tumor, with leaf-like architecture. Stromal projections can be lined by any benign or mildly atypical Mullerian-type epithelia, with or without squamous metaplasia. Intraglandular stromal protrusions are characteristic.^10^

The stroma is typically more cellular and condensed ("collaring") around the glands.^4^ The stroma is usually low-grade and of endometrial stromal or fibroblastic type.^10^ Histopathologically, this case also had similar findings like phyllodes appearance and cuffing of stromal cells around glands. WHO defines adenosarcoma as having a stromal mitotic activity of two or more mitotic figures/10 high power fields, as suggested by Clement and Scully.^2^ Histopathological examination in this case also showed 2-3/10 HPF.

Management is usually done by total hysterectomy and bilateral salpingo-oophorectomy. Negative prognostic factors include the presence of myometrial invasion, sarcomatous overgrowth, lymphovascular invasion, necrosis, and the presence of heterologous elements.^1^ Uterine adenosarcomas are capable of local recurrence, but lymph node and distant metastases are rare. Radiotherapy is not recommended, and there is only limited evidence for the use of neo-/adjuvant or adjuvant chemotherapy and hormonal therapy.^4^ Our patient also underwent a total abdominal hysterectomy with bilateral salpingo-oophorectomy. Uterine adenosarcomas can recur locally in up to 30% of cases, particularly in the vagina; recurrences can be early or late. The presence of deep myometrial invasion is a risk factor for recurrence. Metastatic disease is usually associated with tumors exhibiting sarcomatous overgrowth, and the prognosis is poor.^4^

In conclusion, uterine adenosarcoma is a rare biphasic malignant mesenchymal tumor mostly seen in postmenopausal women, which may be confused as carcinoma of the cervix both clinically and radiologically if the tumor protrudes from the cervix.
